# Development of clinical decision support for patients older than 65 years with fall-related TBI using artificial intelligence modeling

**DOI:** 10.1371/journal.pone.0316462

**Published:** 2025-02-03

**Authors:** Biche Osong, Eric Sribnick, Jonathan Groner, Rachel Stanley, Lauren Schulz, Bo Lu, Lawrence Cook, Henry Xiang

**Affiliations:** 1 Center for Pediatric Trauma Research and Center for Injury Research and Policy, The Abigail Wexner Research Institute at Nationwide Children’s Hospital, Columbus, Ohio, United States of America; 2 Division of Pediatric Neurosurgery, Nationwide Children’s Hospital, Columbus, Ohio, United States of America; 3 Department of Pediatrics, The Ohio State University College of Medicine, Columbus, Ohio, United States of America; 4 Division of Pediatric Surgery, Nationwide Children’s Hospital, Columbus, Ohio, United States of America; 5 Division of Pediatric Emergency Medicine, Nationwide Children’s Hospital, Columbus, Ohio, United States of America; 6 Division of Biostatistics, College of Public Health, The Ohio State University, Columbus, Ohio, United States of America; 7 Pediatric Critical Care, University of Utah School of Medicine, Salt Lake City, Utah, United States of America; National Cerebral and Cardiovascular Center: Kokuritsu Junkankibyo Kenkyu Center, JAPAN

## Abstract

**Background:**

Older persons comprise most traumatic brain injury (TBI)-related hospitalizations and deaths and are particularly susceptible to fall-induced TBIs. The combination of increased frailty and susceptibility to clinical decline creates a significant ongoing challenge in the management of geriatric TBI. As the population ages and co-existing medical conditions complexify*, so does the need to improve the quality of care for this* population. Utilizing early hospital admission variables, this study will create and validate a multinomial decision tree that predicts the discharge disposition of older patients with fall-related TBI.

**Methods:**

From the National Trauma Data Bank, we retrospectively analyzed 11,977 older patients with a fall-related TBI (2017–2021). Clinical variables included Glasgow Coma Scale (GCS) score, intracranial pressure monitor use, venous thromboembolism (VTE) prophylaxis, and initial vital signs. Outcomes included hospital discharge disposition re-categorized into home, care facility, or deceased. Data were split into two sets, where 80% developed a decision tree, and 20% tested predictive performance. We employed a conditional inference tree algorithm with bootstrap (B = 100) and grid search options to grow the decision tree and measure discrimination ability using the area under the curve (AUC) and calibration plots.

**Results:**

Our decision tree used seven admission variables to predict the discharge disposition of older TBI patients. Significant non-modifiable variables included total GCS and injury severity scores, while VTE prophylaxis type was the most important interventional variable. Patients who did not receive VTE prophylaxis treatment had a higher probability of death. The predictive performance of the tree in terms of AUC value (95% confidence intervals) in the training cohort for death, care, and home were 0.66 (0.65–0.67), 0.75 (0.73–0.76), and 0.77 (0.76–0.79), respectively. In the test cohort, the values were 0.64 (0.62–0.67), 0.75 (0.72–0.77), and 0.77 (0.73–0.79).

**Conclusions:**

We have developed and internally validated a multinomial decision tree to predict the discharge destination of older patients with TBI. This tree could serve as a decision support tool for caregivers to manage older patients better and inform decision-making. However, the tree must be externally validated using prospective data to ascertain its predictive and clinical importance.

## Introduction

Traumatic brain injury (TBI) remains a major cause of death and disability in the United States. Recent statistics approximate that 586 TBI-related hospitalizations and 190 TBI-related deaths occur per day [1]. Adults aged ≥ 75 years comprise the majority of TBI-related hospitalizations (32%) and deaths (28%), primarily due to higher infirmity and susceptibility to in-hospital deterioration and poor outcomes [2–4]. The combination of increased frailty and susceptibility to clinical decline creates a significant ongoing challenge in the management of geriatric TBI. As the number of older citizens and the complexity of their co-existing medical conditions increases nationally, so does the need to improve the quality of care for this population.

The biological processes and aftermath of TBI are unclear and differ for each individual, even within a standardized treatment regime. This heterogeneity makes it difficult to accurately predict a patient’s eventual degree of neurologic and functional recovery. Such knowledge is crucial for the families of TBI patients, as it drives goals-of-care conversations surrounding treatment decisions. Accurate prognostic information may also help families prepare financially and mentally for role changes during TBI recovery. However, a personalized prediction of such outcomes can be very challenging even for medical providers, as the amount of information required to make an evidence-based prediction about a patient’s outcome is vast [5,6]. This difficulty calls for support from predictive models, which can efficiently learn from large amounts of patient information and make personalized predictions for future patients.

Practical prediction models have been developed to assist medical providers and caregivers in decision-making for TBI patients [7,8]. Some researchers have applied deep learning techniques, which are known to have superior predictive ability [9–11]. However, these models simply provide an outcome probability without actionable decision points, which healthcare providers could leverage to improve patient care due to their black-box nature. Additionally, some studies have limited their focus to univariate analysis, which fails to account for the multitude of complex factors affecting patient outcomes and obfuscates the complex interplay of said factors.

Prediction models like decision trees are valued in the medical sector because of their high interpretability and actionable insights. Their ability to naturally classify patients into sub-groups based on the outcome of interest makes them an appealing target for clinical application [12]. Some studies have gleaned data from decision trees to predict outcomes in TBI patients [13,14]. However, no study has been tailored specifically to predict hospital discharge disposition in patients over 65 years with fall-induced TBI, even though they are the group most frequently and severely affected [3,4]. In addition, most prior TBI studies focused primarily on a binary outcome; in clinical practice, TBI prognosis and outcomes are highly individualized for each patient. This study, therefore, aims to grow and validate a multinomial decision tree to predict hospital discharge disposition (home, care facility, or death) for older patients with fall-related TBI to improve hospital discharge readiness, facilitate early goals-of-care discussions, and support informed decisions making.

## Methods

### Data source

This study retrospectively collected a subset of the National Trauma Data Bank (NTDB), a comprehensive trauma registry managed by the American College of Surgeons, encompassing voluntarily reported cases from over 765 trauma centers across the United States [15]. For the most recent years included in this study (2017–2021), information on older TBI patients (age 65 and above) with a fall mechanism was identified and analyzed. At the emergency department (ED), patients who were sent to jail, institutional care, mental health facilities, home with or without services, left against medical advice, transferred to another hospital, or expired were excluded. Patients were also excluded if they left the hospital against medical advice, discontinued care, transferred to court, law enforcement, psychiatric hospital, or to another type of institution not defined elsewhere.

### Variable consideration

**Fig 1:**
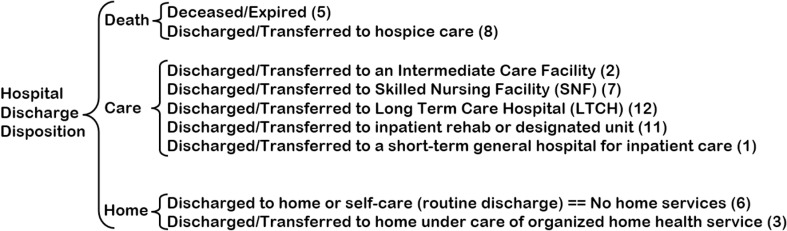
Re-categorization of hospital discharge disposition.

The main objective of this study was to identify patients’ discharge destinations after they left the hospital. To enable a meaningful comparison, the original patients’ discharge disposition was re-categorized into three levels: home, care facility (henceforth called care), and death, as illustrated in Fig 1. Before data analysis, the re-categorization was discussed and agreed upon with a board-certified senior neurosurgeon with nine years of experience in TBI (senior author, EAS). Our subset version of the NTDB considered five main variable groups: socio-demographics, comorbid conditions, brain injury, vital signs, and treatment. However, variables with more than 5% missing values were excluded to prevent excessive data reduction, as all patients without complete information were removed before the tree-growing process. The rationale for focusing on early-obtained variables was to have a model to assist in decision-making early on during a patient’s admission.

### Statistical analysis

A split-sample approach was employed to partition the subset of NTDB data into training (80%) and test (20%) cohorts. The training cohort was used to grow the decision tree, and the test cohort was reserved for assessing predictive performance through metrics including sensitivity, specificity, the area under the receiver operating characteristic curve (AUC), and calibration plots. Descriptive statistics and graphical analyses were conducted to evaluate variable distributions, assess the extent of missingness, and identify outliers or erroneous values. The decision tree was developed using a **conditional inference tree (CIT)** algorithm, chosen for its effectiveness in determining a predictor’s true effect on the outcome when simultaneously considering multiple variables. Unlike traditional decision tree algorithms, the CIT approach employs permutation-based significance tests at each node and corrects for multiple testing (Bonferroni), ensuring unbiased variable selection toward predictors with more levels or higher variance [16]. We used the bootstrap resampling technique (B = 100) to enhance stability and generalizability. Optimal hyperparameters, including tree depth and minimum splitting criterion, were identified through a grid search based on accuracy. All analyses were performed with R software version 4.3.0 [17], and a p–value *< * 0.01 was considered statistically significant.

## Results

Information from 11,977 patients over 65 years who experienced a fall-related TBI between 2017 and 2021 was analyzed from the NTDB. We combined the information extracted over the five years and then split it into two cohorts. The first cohort trained the model and comprised 80% of the total population (n of 9,181), while the second cohort tested the model performance and comprised the remaining 20% (n of 2,296). The distribution of patients within the three levels of the outcome in the training cohort was 3,509 patients in the death group (38.2%), 4,099 patients in the care group (44.6%), and 1,573 patients in the home group (17.1%). Patients with incomplete information were not analyzed further, which reduced the size of the training and test cohorts to 8,025 and 2,011, respectively.

Tables 1 and 2 show the considered variable distributions for the training and test sets. Categorical variables show the number of patients in each category with the respective percentages, while continuous variables show the mean with standard deviation (sd) in the train and test data. The median age of patients was 76 (65 to 89) years. Age, vital signs, and injury severity exhibited very weak linear relationships (S1 Fig supplemental material), with a maximum positive and negative correlation coefficient of 0.10 and -0.14, respectively, between the three groups of variables. The coefficient of variation for age was 10%, while that of injury severity variables was 40%, and vital signs < 30%.

**Table 1:** Patient socio-demographic and comorbid conditions on the training and test dataset.

Variable	Levels	Train	Test
Age (years)	Mean (sd)	76.5 (6.6)	76.6 (6.7)
Sex	Male (1)	5469 (59.8%)	1370 (59.9%)
	Female (2)	3669 (40.2%)	917 (40.1%)
	*Missing*	45 (0.5%)	09 (0.4%)
Substance Screening	None	7990 (88.0%)	2017 (88.7%)
	Alcohol	403 (4.4%)	99 (4.4%)
	Smoking	521 (5.7%)	126 (5.5%)
	Both	167 (1.8%)	31 (1.4%)
	*Missing*	100 (1.1%)	23 (1.0%)
Hospital Type	For-profit (1)	8164 (89.0%)	2036 (88.7%)
	Non-profit (2)	998 (10.9%)	258 (11.3%)
	Government (3)	11 (0.1%)	01 (0.1%)
	*Missing*	08 (0.1%)	01 (0.1%)
Anticoagulant Therapy	Yes (1)	3944 (43.3%)	1023 (44.9%)
	No (2)	5168 (56.7%)	1255 (55.1%)
	*Missing*	62 (0.8%)	18 (0.8%)
Diabetes	Yes (1)	3530 (38.8%)	919 (40.4%)
	No (2)	5571 (61.2%)	1356 (59.6%)
	*Missing*	80 (0.9%)	21 (0.9%)
Functional Dependency	Yes (1)	2747 (30.2%)	697 (30.7%)
	No (2)	6342 (69.8%)	1577 (69.3%)
	*Missing*	92 (1.0%)	22 (1.0%)
Hypertension and Renal Problems	None	3529 (38.8%)	937 (41.2%)
	Hypertension	5292 (58.3%)	1273 (56.0%)
	Renal	43 (0.5%)	14 (0.6%)
	Both	220 (2.4%)	50 (2.2%)
	*Missing*	89 (1.0%)	22 (1.0%)

**Table 2:** Brain injury and major treatment characteristics on the training and test dataset.

Variable	Levels	Train	Test
Injury Severity Score	Mean (sd)	20.8 (8.4)	20.8 (8.2)
Systolic Blood Pressure	Mean (sd)	153.3 (30.3)	153.7 (29.6)
	*Missing*	187 (2.0%)	53 (2.3%)
Total GCS	Mean (sd)	11.2 (4.5)	11.4 (4.4)
	*Missing*	380 (4.1%)	109 (4.7%)
Pulse Rate	Mean (sd)	84.8 (19.5)	84.6 (19.2)
	*Missing*	158 (1.7%)	52 (2.3%)
Pulse Oximetry	Mean (sd)	96.8 (4.3)	96.7 (4.9)
	*Missing*	236 (2.6%)	75 (4.7%)
Respiratory Rate	Mean (sd)	18.5 (4.9)	18.1 (4.7)
	*Missing*	307 (3.3%)	83 (3.6%)
GCS Eye	No eye movement (1)	2120 (24.1%)	481 (22.1%)
	Pain response (2)	404 (4.6%)	101 (4.6%)
	Verbal response (3)	729 (8.3%)	200 (9.2%)
	Spontaneous (4)	5527 (62.9%)	1395 (64.1%)
	*Missing*	401 (4.4%)	119 (5.2%)
GCS Verbal	No verbal response (1)	2497 (28.4%)	578 (26.6%)
	Incomprehensible (2)	535 (6.1%)	146 (6.7%)
	Inappropriate (3)	317 (3.6%)	90 (4.1%)
	Confused (4)	2202 (25.1%)	545 (25.0%)
	Oriented (5)	3227 (36.8%)	818 (37.6%)
	*Missing*	403 (4.4%)	104 (4.5%)
GCS Motor	No motor response (1)	1433 (16.3%)	330 (15.2%)
	Extension to pain (2)	146 (1.7%)	39 (1.8%)
	Flexion to pain (3)	174 (2.0%)	41 (1.9%)
	Withdrawal (4)	686 (7.8%)	172 (7.9%)
	Localizing pain (5)	1045 (11.9%)	238 (10.9%)
	Motor response (6)	5290 (60.3%)	1357 (62.3%)
	*Missing*	407 (4.4%)	108 (4.7%)
AIS Severity	Moderate (2)	951 (10.4%)	231 (10.1%)
	Serious (3)	4068 (44.3%)	1003 (43.7%)
	Severe (4)	1235 (13.5%)	309 (13.5%)
	Critical (5)	2927 (31.9%)	753 (32.8%)
ED Discharge Disposition	Floor bed (1)	609 (6.6%)	159 (6.9%)
	Observation unit (2)	40 (0.4%)	12 (0.5%)
	Step-down unit (3)	368 (4.0%)	88 (3.8%)
	Operating room (7)	2051 (22.3%)	495 (21.6%)
	Intensive Care Unit (8)	6113 (66.6%)	1542 (67.2%)
Intracranial Pressure Monitoring	No (0)	8061 (87.8%)	2002 (87.2%)
	Yes (1)	1120 (12.2%)	294 (12.8%)
Life Support Treatment Withdrawal	Yes (1)	2429 (27.4%)	602 (26.2%)
	No (2)	6445 (72.6%)	1611 (70.2%)
	*Missing*	307 (3.3%)	83 (3.6%)
Venous Thromboembolism Prophylaxis Type	Heparin (1)	793 (8.9%)	211 (9.4%)
	None (5)	3931 (44.1%)	1006 (44.9%)
	LMWH (6)	2229 (25.0%)	553 (24.7%)
	Other (10)	106 (1.2%)	30 (1.3%)
	UH (11)	1861 (20.9%)	442 (19.7%)
	*Missing*	261 (2.8%)	54 (2.4%)
Discharge	Death	3509 (38.2%)	881 (38.4%)
	Care	4099 (44.6%)	1028 (44.8%)
	Home	1573 (17.1%)	387 (16.9%)

**AIS**, Abbreviated Injury Scale; **ED**, emergency department; **GCS**, Glasgow Coma Scale; **LMWH,** Low Molecular-Weight Heparin; **UH**, Unfractionated Heparin.

More males, 5469 (59.8%) comprised the population than females, 3669 (40.2%), with most patients being hypertensive (58.3%). Diabetic patients accounted for 38.8% of the total population, but only approximately 12% of patients were habitual smokers or alcohol consumers. In the ED, 66.6% of the patients were admitted to the intensive care unit, while 22.3% went to the operating room. Only a small number of patients (< 11%) were admitted to the floor and step-down unit, while < 1% were placed in the observation unit. VTE prophylaxis treatment was not administered to nearly half of the patient population (43.1%), while for those who did receive VTE prophylaxis treatment, low-molecular-weight heparin (25.0%) and unfractionated heparin (20.9%) were the most commonly used treatments. Only 12.2% of patients received cerebral monitoring, while 27.4% were taken off life-supporting treatment.

**Fig 2:**
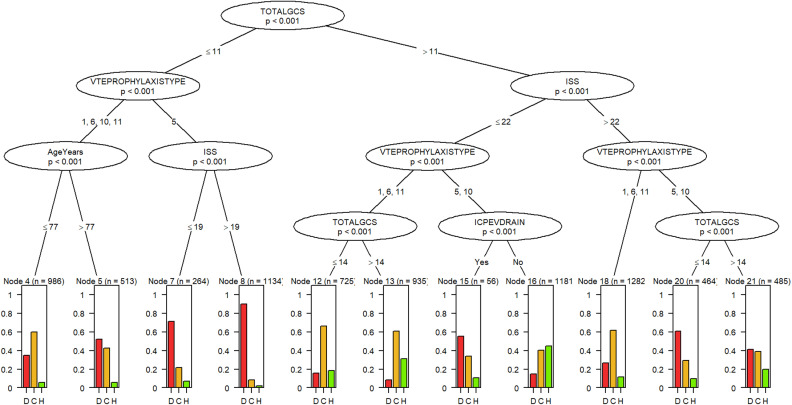
The developed multinomial decision tree to predict discharge disposition in older head injury patients with a fall mechanism. The oval structures, i.e., nodes, are patient characteristics (variables) arranged in descending order of importance. The lines are the branches that connect the variables and hold the conditions for splitting the data into smaller groups of patients with similar characteristics. The rectangular structures at the bottom of the tree are called leaf or decision nodes. The values at the top of the decision nodes indicate the number of patients in that node, and the p-values in the oval structures indicate the significance of the split. To utilize the decision tree as a decision-making tool, one would begin from the topmost node and follow the branches that correspond to the patient’s condition in a continuous flow till the leaf node, then read the patient’s probability of being discharged to either a care facility (yellow), home (green), or death (red). ISS Injury Severity Score, **ICPEVDRAIN:** ICP Monitoring, **VTEPROPHYLAXISTYPE:** Venous Thromboembolisms (VTE) prophylaxis type, **1** = Heparin, **5** = None, **6** = Low-Molecular-Weight Heparin, **10** = Others and **11** = Unfractionated Heparin.

Fig 2 shows the developed decision tree with the optimal bootstrap hyperparameters (S2 Fig supplemental material) to predict the hospital discharge disposition of patients over 65 with a fall-related TBI. The tree diagram shows the characteristics of patients in order of importance. The lines between the characteristics show how the data splits into smaller groups of patients with similar characteristics. The decision node at the bottom of the tree gives the probability of being discharged to either a care facility (gray), home (white), or death (black). To utilize the tree as a decision-making tool, one would begin with the most pivotal patient characteristic and follow the tree splits corresponding to the patient’s condition in a continuous flow until the decision node, where the discharge probability of the patient is determined for each disposition.

Based on the decision tree of the five groups of variables considered, patients’ age, TBI severity, intracranial pressure (ICP) monitoring, and VTE prophylaxis treatment were the most significant predictors of hospital discharge disposition in older patients after a fall-related TBI. Generally, TBI severity variables separated the patient population into low, moderate, and high-risk of poor outcome prognosis in terms of death or transfer to a care facility. Patients with a GCS score of ≤  11 were categorized as having a high risk of poor outcomes, while those with a GCS score of > 11 and an ISS > 22 had a moderate risk of poor outcomes. Patients with a GCS score of > 11 but an ISS <  22 fell under the low-risk category (Equation 1).

$$\mathrm{Risk}\;\mathrm{Groups}= \{\begin{array}{l} \mathrm{GCS}£11\;-\mathrm{High}\;\mathrm{Risk}\\ \mathrm{GCS}>11\;\&\;\mathrm{ISS}\;>\;22-\mathrm{Moderate}\;\mathrm{Risk}\\ \mathrm{GCS}>11\;\&\;\mathrm{ISS}\;£\;22-\mathrm{Low}\;\mathrm{Risk} \end{array}$$ (1)

The tree shows that VTE prophylaxis significantly reduced a patient’s chance of death for all risk groups. However, for the high-risk group, age was a significant split factor for patients who did receive VTE prophylaxis treatment, and patients younger than 77 had a somewhat better prognosis than their older counterparts based on the likelihood of death or being discharged to a care facility. In the low-risk group, ICP monitoring was a crucial split factor for patients not treated with VTE prophylaxis (5) or who received other forms of VTE prophylaxis treatment (10). Patients who did not undergo external ventricular drain (EVD) had a higher probability of returning home. In contrast, the chances of death for patients who had EVD placement were relatively very high. However, the likelihood of being discharged to a care facility was comparable in both groups.

To evaluate the tree’s performance, we first extracted the predicted probabilities of each level of the outcome: death, care, and home. Then, their respective AUC, accuracy, sensitivity, and specificity values were computed, as shown in Table 3. For the training data, the AUC (95% confidence interval) values for death, care, and home were 0.66 (0.65-0.67), 0.75 (0.73-0.76), and 0.77 (0.76-0.79), respectively (S3 Fig supplemental material). In the test data, the values were 0.64 (0.62-0.67), 0.75 (0.72-0.77), and 0.77 (0.73-0.79). The tree showed better performance in identifying patients within the different outcome levels. The sensitivity rate for deceased patients was 79%, while for patients discharged to a care facility or home, the sensitivity rates were 71% and 74%, respectively. In contrast, the specificity rate for all three outcome levels was relatively low, with 65% and 60% for home and care, while the specificity rate for the deceased level was just 50%. These results were similar in the training cohort.

**Table 3:** The tree’s accuracy, AUC, sensitivity, and specificity values on the train and test data.

Data	Discharge	AUC (95% CI)	Cutoff	Accuracy	Sensitivity	Specificity
**Train**	Care	0.75 (0.73–0.76)	0.5	0.65	0.71	0.61
	Death	0.66 (0.65–0.67)	0.4	0.64	0.80	0.50
	Home	0.77 (0.76–0.79)	0.1	0.68	0.77	0.66
**Test**	Care	0.75 (0.72–0.77)	0.5	0.65	0.71	0.60
	Death	0.64 (0.62–0.67)	0.4	0.62	0.79	0.50
	Home	0.77 (0.73–0.79)	0.1	0.67	0.74	0.65

AUC, area under the curve; CI, confidence interval

**Fig 3:**
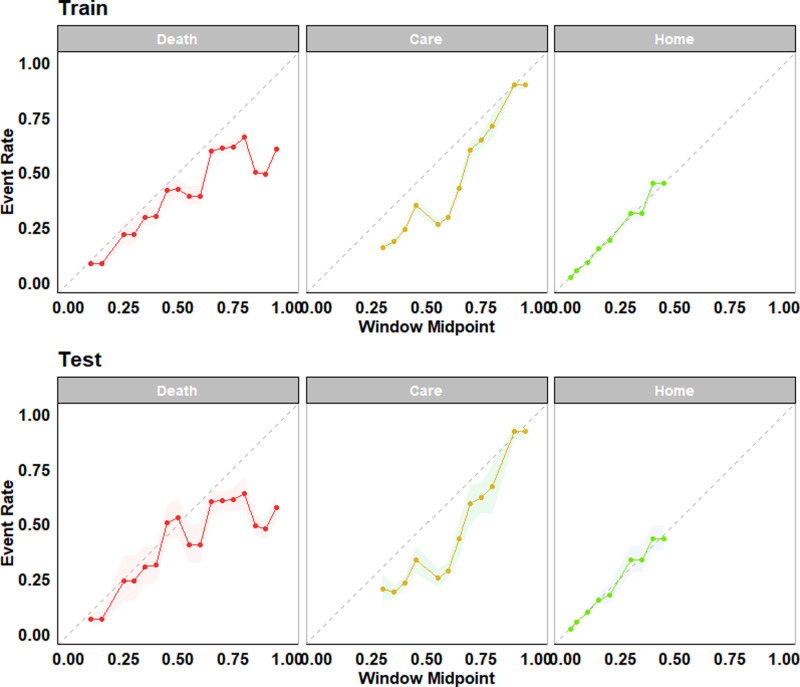
Calibration plots of the grown tree on the train (top) and test (bottom) data by hospital discharge disposition outcome (care, death, home). The gray dashed line represents an ideal model, and solid lines represent the tree’s performance with 95% confidence intervals.

Fig 3 displays the calibration plot, which shows the similarity between a model’s predicted probabilities and the observed frequencies in the data. Most points are closer to the gray diagonal line, particularly for patients discharged home, which shows the tree’s predictive ability. The tree consistently underestimated the likelihood of a patient being discharged to a care facility, while its ability to predict which patients are likely to be deceased varied more so for larger probabilities.

## Discussion

Recovery from a TBI is a fascinatingly complicated and complex process, with multiple factors in interplay. The inherent uncertainty regarding an individual’s clinical progress over time increases clinicians’ challenges in providing accurate prognostic information to patients and their families. To assist in illuminating our understanding of this process, we aimed to develop a multinomial decision tree to predict the discharge destination of older patients suffering from fall-related TBIs using early-derived hospital admission variables. Our findings showed that the severity of TBI and the medical treatment, especially VTE prophylaxis, were the most significant factors in determining an older patient’s discharge destination.

After a TBI, a patient’s neurologic status is often evaluated using a clinical scale, such as the GCS score [4]. However, some authors have expressed concerns regarding the limitations and potential biases of the GCS score [4,18], especially for the geriatric population, who often exhibit higher initial GCS scores compared to adults with the same injury severity [4,19–21] but with poorer outcomes [22]. Some authors argue that alcohol and drug intoxication may also confound the GCS score of trauma patients [23]. Others suggest that in the setting of polypharmacy for multiple medical comorbidities such as chronic pain, GCS scoring in older trauma patients may not be accurate [4,24,25]. Nonetheless, studies have also shown that GCS scores have some predictive ability, more so for mortality [26–28]. For example, Caterino et al. [29] found that higher GCS scores in older patients improved their model’s predictive ability for different outcomes, including neurological intervention and mortality.

Based upon our decision tree, the GCS score was the most important predictive factor for older patients’ hospital discharged disposition. Generally, the GCS score is a comatose criterion associated with neurologic injury severity [30], and mortality rates increase with decreasing GCS scores [7]. The tree associates patients with GCS scores of ≤11 with a high risk of an unfavorable outcome of death, which is consistent with previously published literature [22]. While we recognize the limitations of utilizing a single variable as a predictive factor, these findings emphasize the importance of recognizing poor initial neurologic status as a serious indicator of unfavorable outcomes in older TBI patients. This information may help initiate early goals-of-care discussions with family members, which has been shown to significantly reduce symptoms of stress, post-traumatic anxiety, and depression in surviving family members [31]. Based on The American College of Surgeons Trauma Quality Improvement Program, palliative care best practices guidelines recommend trauma teams initiate goals of care discussions within 72 hours of admission for patients with potentially life-threatening or disabling injuries as well as preinjury serious illness [32].

Traumatic injuries, including TBI, may cause the body to enter a state of hypercoagulability or venous stasis, making VTE prophylaxis crucial in the management of TBI patients [33]. According to the 2020 Western Trauma Association guidelines, pharmacologic prophylaxis should be given to nearly all TBI patients within 72 hours of injury [34]. However, some studies have shown that early use of VTE chemoprophylaxis in TBI patients may increase the risk of intracranial hemorrhage, making some physicians hesitant to prescribe [35]. On the other hand, delaying the administration of VTE prophylaxis to patients who require it can increase the risk of developing VTE [33,36], resulting in health complications [37,38]. Therefore, it can be challenging to determine the optimal and safe timing of VTE prophylaxis in TBI patients in the clinical setting [33,36]. Based on our decision tree, patients who did not receive VTE prophylaxis had a much higher probability of death than being discharged home or to a care facility. Our findings reinforce recent trauma-based literature and underscore the importance of early VTE prophylaxis administration despite the perceived risks of increased bleeding.

ICP monitoring is integral to TBI management, especially in severe injury, resulting in a lack of a reliable neurologic exam [39–41]. The gold standard is to place a temporary device connecting the brain to a pressure transducer outside the body with a catheter [39,40]. This procedure may be achieved via the placement of a “bolt,” intra-parenchymal pressure monitor, or an external ventricular drain (EVD), which has the dual use of cerebrospinal fluid drainage and ICP monitoring. ICP monitoring allows timely interventions to alleviate changes in the neurologic exam resulting from increased ICP, theoretically minimizing ongoing brain damage [39–41]. However, studies show mixed evidence on the benefit of ICP monitoring, possibly due to differences in analytical approach and the patient population of these studies [4]. Nonetheless, our study found that for patients in the low-risk branch of the tree (GCS *>* 11 and ISS ≤ 22) who did not receive VTE prophylaxis (5) or received other forms of VTE prophylaxis (10), cerebral monitoring was not beneficial based on the outcome. The tree showed that patients who had an EVD placement were about four times more likely to die (65%) compared to patients without EVD placement (17%), who also had the highest chance of being discharged home. However, ICP monitor placement is only indicated for patients exhibiting a GCS of ≤ 8 per the Brain Trauma Foundation Guidelines. Therefore, despite their initial benign GCS and ISS, this finding likely represents a sub-group of patients who experienced rapid neurologic deterioration during their clinical course and thus were fated to an unfavorable outcome (death) regardless of intervention.

### Limitations

The strength of our study lies in the tree’s ability to simultaneously classify patients into the three main hospital discharge dispositions after a TBI, which may assist family members in better preparing for discharge needs. Nonetheless, our study is hardly devoid of limitations, the primary of which derives from using retrospective data to build our predictive model. As with all retrospective data, this data contained missing patient information and inherent variability outside our control, which could partly explain the tree’s suboptimal performance on some outcome levels. For instance, as Stanley et al. [42] showed, non-patient-specific factors like geographic region, hospital size, trauma level, and teaching status may also influence the discharge destination of TBI patients. Moreover, using only baseline severity scores implies the tree can only partially capture the dynamic nature of the patient’s condition, as some patients’ health status can change significantly after admission, requiring a reassessment of their risk group. However, we utilized baseline information to have a model that can be applied upon a patient’s admission to provide an early prognosis, necessitating any adjustment. In addition, studies have demonstrated that baseline indicators such as admission vitals and injury severity variables like Glasgow Coma Scale (GCS) scores are among the strongest predictors of outcomes in TBI patients [43,44].

## Conclusion

We have developed and internally validated a decision tree utilizing early admission variables from a nationwide database to predict hospital discharge disposition in patients over 65 years with a fall-related TBI. The visual representation of the grown decision tree provides valuable insights for patients in the different leaf nodes, which medical care providers and TBI patients’ families can leverage for better patient management or informed decision-making. However, an external validation of the tree utilizing a prospective patient cohort is warranted to ascertain its predictive performance and clinical importance.

## Supporting information

S1 Fig Spearman correlation coefficient of age, injury severity, and vital signs with their histogram and scatter plot. (TIF)

S2 Fig Bootstrap (B = 100) output for selecting optimal parameters for growing the multinomial decision tree. (TIF)

S3 Fig The area under the curve (AUC) of the grown multinomial decision tree on the training and test data, respectively, for the different hospital discharge dispositions. (TIF)
