# Supplementary figures and images for: Development of clinical decision support for patients older than 65 years with fall-related TBI using artificial intelligence modeling
Source: PLoS One. 2025 Feb 3;20(2):e0316462. doi: 10.1371/journal.pone.0316462 (PMC11790116; doi:10.1371/journal.pone.0316462)

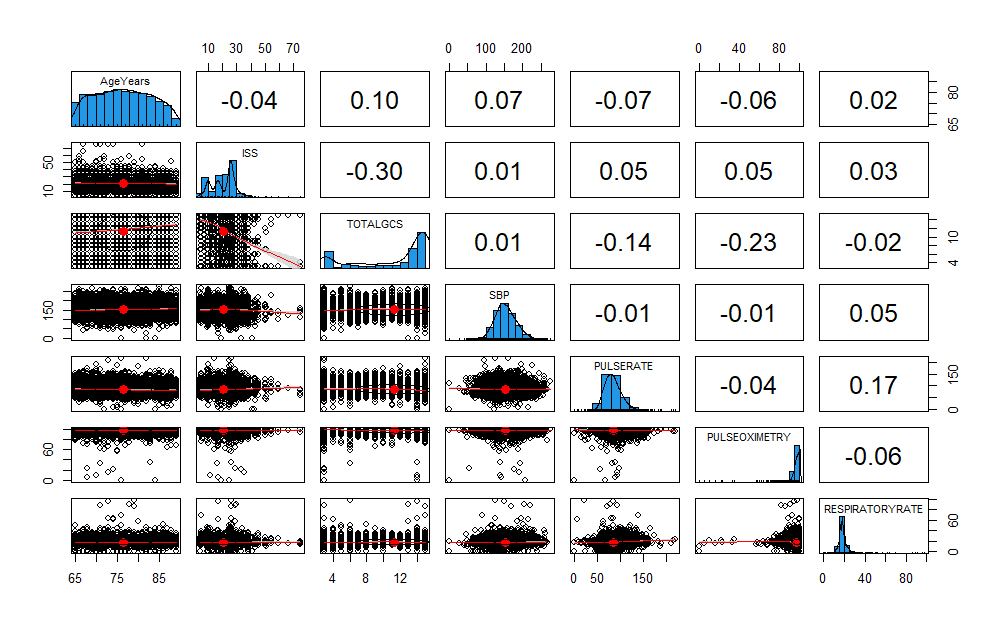

Supplement: S1 Fig — (TIF) [file pone.0316462.s001.tif]

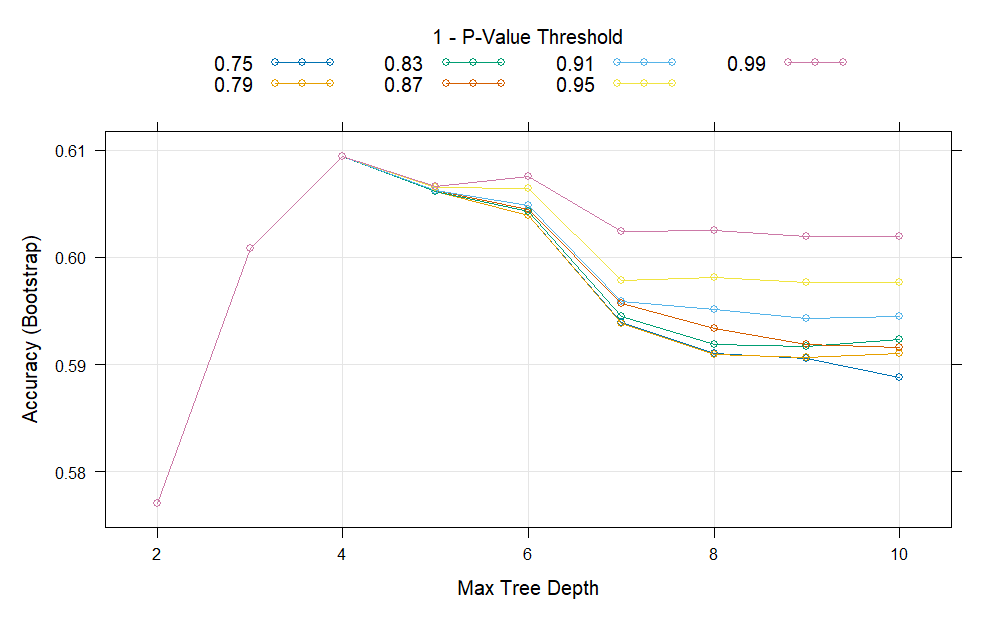

Supplement: S2 Fig — (TIF) [file pone.0316462.s002.tif]

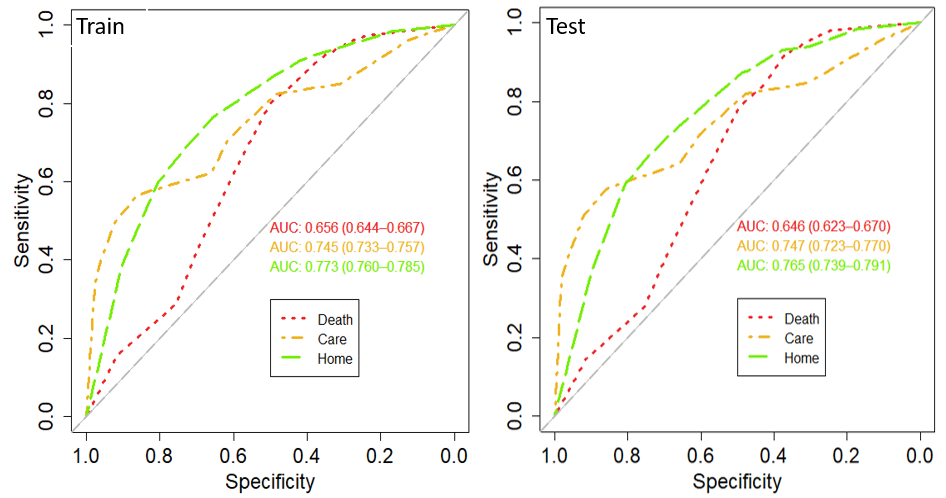

Supplement: S3 Fig — (TIF) [file pone.0316462.s003.tif]
